# Near-universal marriage, early childbearing, and low fertility: India’s alternative fertility transition

**DOI:** 10.4054/DemRes.2023.48.34

**Published:** 2023-06-27

**Authors:** Narae Park, Sangita Vyas, Kathleen Broussard, Dean Spears

**Affiliations:** 1 Population Research Center and Population Wellbeing Initiative at the University of Texas at Austin, USA.; 2 Hunter College and CUNY Institute for Demographic Research at the City University of New York, Population Wellbeing Initiative at the University of Texas at Austin, USA, and r.i.c.e..; 3 Department of Sociology at the University of South Carolina, Population Wellbeing Initiative at the University of Texas at Austin, USA.; 4 Department of Economics, Population Research Center and Population Wellbeing Initiative at the University of Texas at Austin, USA, r.i.c.e., and IZA, Bonn, Germany.

## Abstract

**OBJECTIVE:**

To compare fertility in India to both low-to-middle-income and high-income countries (LMICs and HICs) and describe the patterns that have accompanied India’s transition to low fertility.

**METHODS:**

We use data from the Demographic and Health Surveys (DHS), the United Nations (UN), and the Organisation for Economic Co-operation and Development (OECD) to observe factors associated with fertility decline in 36 Indian states and 76 countries.

**RESULTS:**

Although fertility in India has declined to levels similar to HICs, women’s entry into marriage and initiation of childbearing are more in line with patterns found in LMICs. The vast majority of women in India (97%) are married by age 30, and their average age at first birth is only 21.3 years old. In spite of these patterns, average fertility has declined in India as a result of earlier termination of childbearing. Among more recent cohorts, fewer women progressed to higher-order births and about half of women obtained a sterilization by age 35.

**CONCLUSIONS:**

India has reached low fertility by mechanisms outside the traditional indicators of fertility decline. In contrast to countries that have achieved low fertility through delayed age at first birth, women in India have continued to enter unions and bear children early, lowered their age at last birth, and increasingly ended their fertility via sterilization following the birth of two children.

**CONTRIBUTION:**

Evidence from India reveals an alternative pathway to low fertility, highlighting the limitations of traditional socioeconomic indicators for explaining fertility decline.

## 1. Background

Demographers have long investigated the relationship between development and fertility, broadly attributing fertility decline to material and ideational change in society (Bongaarts and Watkins 1996; Bryant 2007; Cleland and Wilson 1987; Galloway, Hammel, and Lee 1994; Potter, Schmertman, and Cavenaghi 2022; Watkins 1987). In high-income countries (HICs), fertility decline was long correlated with increases in women’s participation in the labor force and higher education (Devaney 1983; Adserà 2004; Sobotka, Beaujuoan, and Van Bavel 2017). As women devoted a greater proportion of their early childbearing years to these pursuits, rates of childlessness and the average age at first birth both increased, contributing to fertility postponement and decline (Abbasi-Shavazi, McDonald, and Hosseini-Chavoshi 2009; Adserà 2004; Devaney 1983; Frejka and Sardon 2006; Yoo 2014).

India will soon surpass China as the most populous country in the world (UN DESA 2022), making it an important place to understand the causes and consequences of fertility decline. Despite continued population growth, fertility has long been declining in the country, with states in the South generally leading the transition (Visaria and Visaria 1994; Guilmoto and Rajan 2005). India recently joined over 80 countries with sub-replacement fertility (UN DESA 2020), reaching a total fertility rate (TFR) of just 2.0 births per woman (India DHS 2019‒2021). Despite this convergence to sub-replacement fertility, India’s journey through demographic transition has occurred with minimal changes in women’s labor force participation (Gietel-Basten, Spears, and Visaria 2021), marriage dynamics (Dommaraju 2013), and family change (Ram 2012), in notable contrast to historical patterns observed in HICs.

In this article, we document additional features of India’s transition to low fertility that highlight its experience compared to other countries. In the first part of our analysis, we show that TFR in India is more closely aligned with HICs at two children per woman, yet the vast majority of women in India get married and start childbearing early, patterns that are more similar to LMICs. The second part of our analysis documents patterns associated with fertility decline in India. In contrast to the processes that have accompanied sub-replacement fertility in HICs, India’s transition to low fertility across cohorts of women has occurred along with only small changes in the proportion of women who marry, age at first marriage, and age at first birth. The decline in fertility across cohorts is, however, associated with substantial reductions in the age at last birth. Relative to older cohorts, women in more recent cohorts have substantially shortened their childbearing careers through the use of female sterilization.

India is not the only country to have reached sub-replacement fertility through pathways that differ from those experienced in Western countries (Lesthaeghe 2014). For example, the close link between cohabitation and fertility postponement observed in Western countries was not observed in East Asia and Latin America (ibid.). India’s experience of transition to low fertility with little change in entry into marriage or initiation of childbearing provides further evidence for the existence of alternative pathways to fertility transition.

## 2. Data and methods

For our cross-country analyses, we compare India to 76 countries. To compare marriage rates, we use data from the UN World Fertility and Marriage Data. To compare age at first birth, we use Demographic and Health Survey (DHS) data for 44 LMICs and Organization for Economic Cooperation and Development (OECD) data for 32 primarily HICs. In these analyses, data on India are from India’s 2019‒2021 DHS. For all countries, data from the most recent survey year is used.

For our within-country analyses, we use data from India’s 1992‒1993, 1998‒1999, 2005‒2006, 2015‒2016, and 2019‒2021 DHS. Combining data from the five rounds allows us to examine changes across cohorts and differences between regions in fertility, ideal fertility, the timing of marriage and initiation of childbearing, and contraceptive use.

For fertility, we study parity at age 30, an age at which the vast majority of women in India have ended their childbearing careers. Studying parity at older ages would substantially limit the sample; we note, however, that when we do examine parity at older ages our findings are similar. For ideal fertility, the DHS asks the respondent how many children she would choose to have in her lifetime. To compare entry into marriage and initiation of childbearing we focus on several factors, including the proportion of women cohabitating by age 30, age at first cohabitation, age at first birth, and age at last birth. Finally, we examine patterns in contraceptive use, and in particular the use and timing of female sterilization by age and parity.

The within-country analyses examine changes across birth cohorts of women in India, and also explore differences across regions. In the analyses that follow we compare four northern states, Bihar, Madhya Pradesh, Rajasthan, and Uttar Pradesh, which have relatively high fertility, to four southern states, Andhra Pradesh, Kerala, Karnataka, and Tamil Nadu, which have relatively low fertility.

## 3. Results

**Figure 1:**
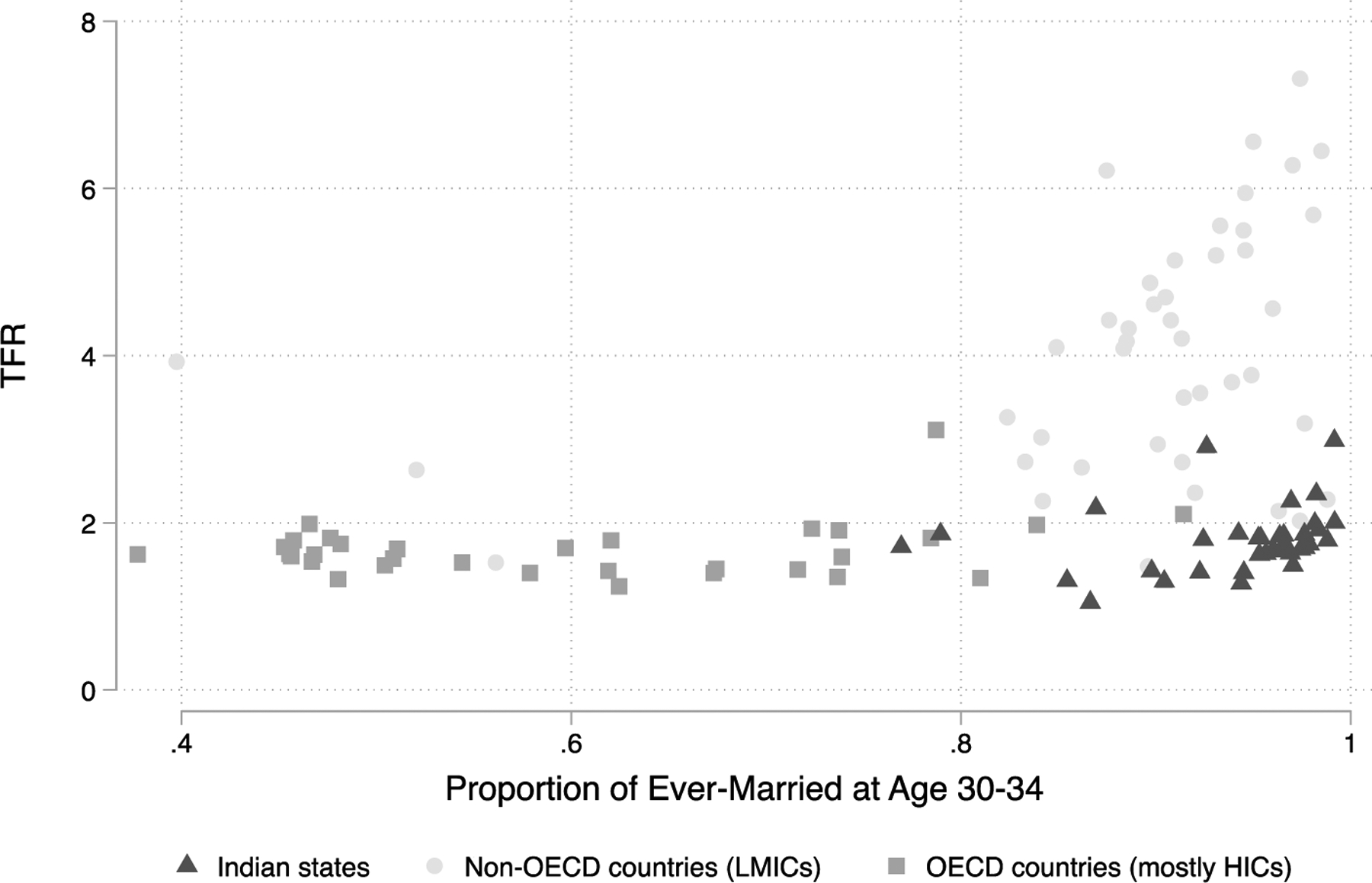
India has low fertility despite a high proportion of married women *Note*: The 36 Indian states are represented by dark gray triangles. Non-OECD countries are represented by light gray circles. OECD countries are labeled as medium gray rectangles. *Source*: UN World Fertility and Marriage for all countries but India and DHS for Indian states.

**Figure 2:**
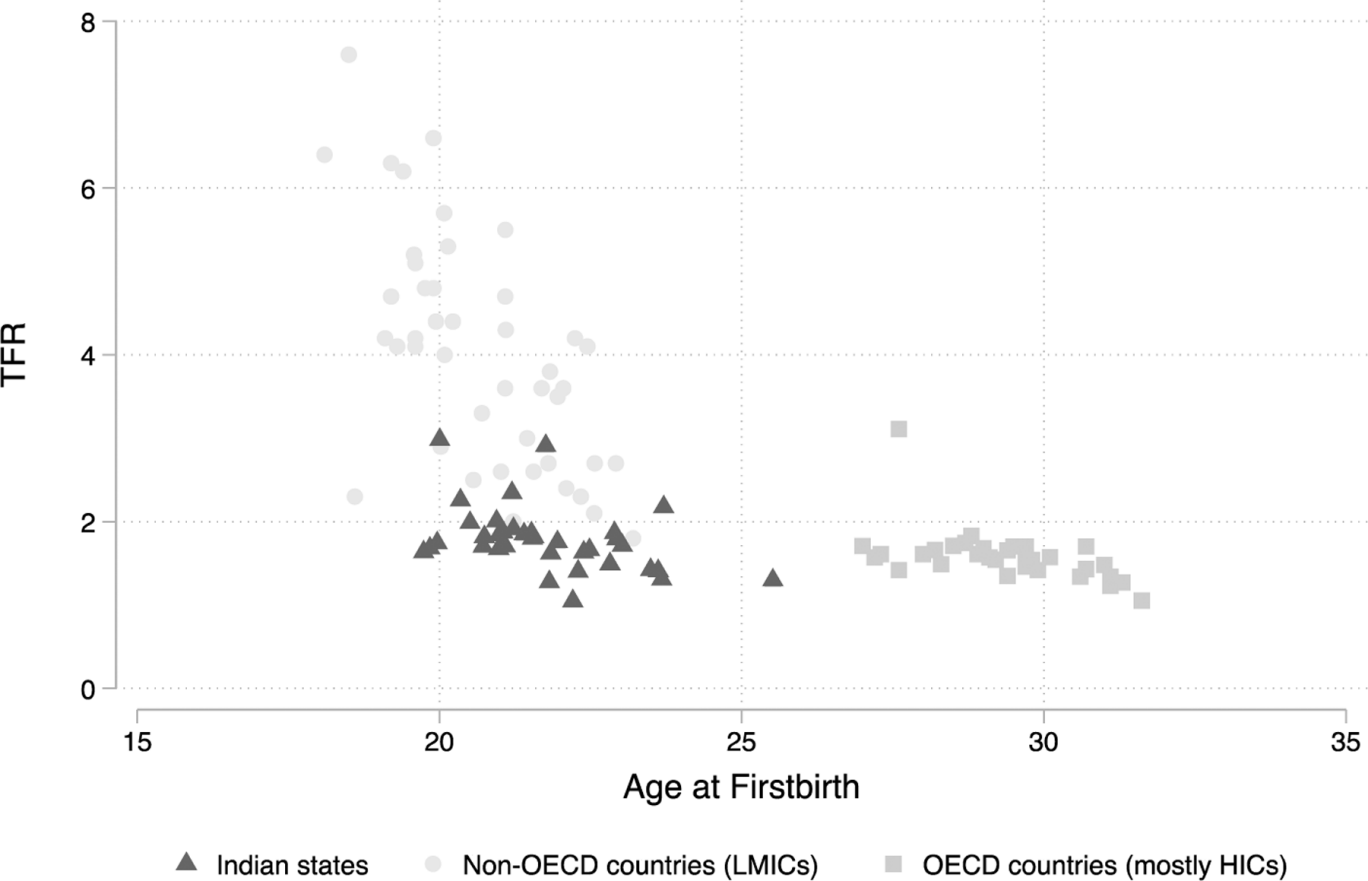
India has low fertility despite a low average age at first birth *Note*: The 36 Indian states are represented by dark gray triangles. Non-OECD countries are represented by light gray circles. OECD countries are labeled as medium gray rectangles. *Source*: OECD Family Database for OECD countries and DHS for non-OECD countries and India.

In Figures 1 and 2 we document India’s low-fertility exceptionalism by comparing Indian states to HICs and other LMICs regarding total fertility, the proportion of women aged 30‒34 ever-married, and average age at first birth. Occupying the space between HICs and LMICs, TFR in India is more closely aligned with HICs at two children per woman, yet the vast majority of women in India get married and start childbearing early, patterns that are more similar to LMICs. In no other country with TFR less than or equal to 2.0 is the proportion of ever-married women as high as in India. Similarly, only one other country has a similarly low TFR and average age at first birth as India. Although there is heterogeneity across Indian states, TFR is below 2.0 in 30 of 36 states, and in all except one, average age at first birth is below 25. These figures demonstrate India’s position as an outlier regarding its low fertility, despite the fact that most women get married and begin childbearing at young ages.

**Figure 3:**
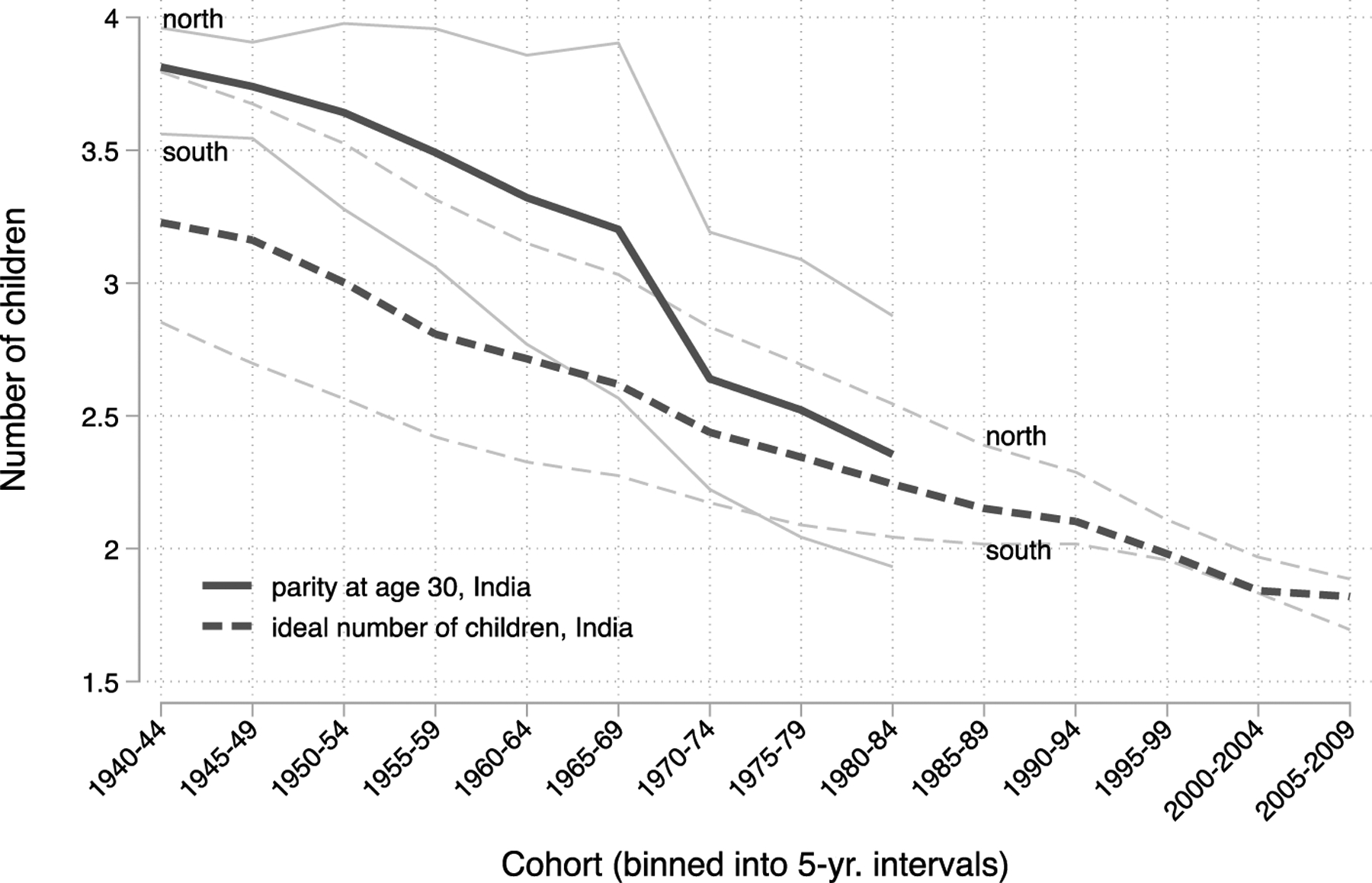
Declining age 30 parity and ideal fertility across cohorts in India *Note*: Black lines are estimates of parity at age 30 and ideal number of children for India as a whole. The gray lines are estimates for the northern and southern states, respectively, and are labeled. *Source*: NFHS 1‒5.

How has the TFR in India declined despite the fact that women still initiate childbearing relatively early by international standards? The remainder of our analysis examines patterns within India and trends across cohorts. We compare four northern states to four southern states, which have historically had relatively high and low fertility, respectively. Figure 3 shows parity at age 30 and ideal fertility across 5-year cohorts of women born between 1940 and 2009, for India as a whole and across regions. Women born in cohorts beginning in 1990 had not reached age 30 by the time they were surveyed. As in the previous figure, there is substantial heterogeneity across Indian states, with southern states having substantially lower fertility than northern states. However, fertility is declining in all states.

Ideal fertility is also declining in India. Like the fertility differences across regions, ideal fertility is lower in southern states. However, among the youngest cohorts, ideal fertility has reached levels below 2.0 in both regions. Among women born in 2000 or later, about 9% stated an ideal of zero, 10% stated an ideal of one, and 72% stated an ideal of two. This demonstrates a low fertility norm that is becoming stronger across cohorts and does not provide any evidence that India’s fertility decline will halt at replacement rate.

**Figure 4:**
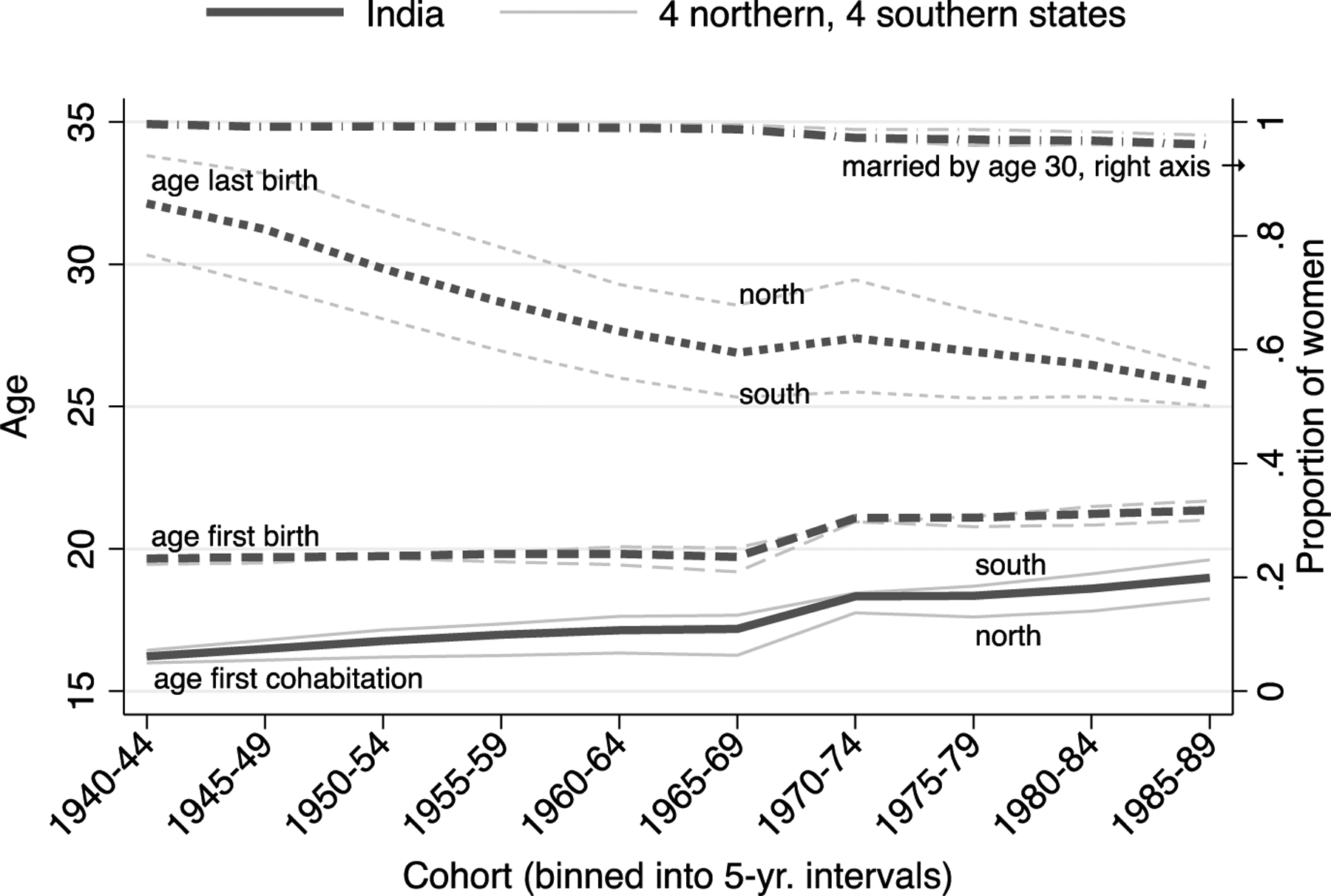
Small increase in age at first birth and large declines in age at last birth across cohorts in India *Note*: Black lines are estimates for India as a whole. Gray lines are estimates for northern and southern states, respectively. The lines for north and south for age at first birth and married by age 30 are not separately labeled because they are so similar to each other. *Source*: NFHS 1‒5.

Is declining fertility associated with differences in family formation and the timing of childbearing? Figure 4 responds to this question by showing, by cohort, the proportion of women that are married by age 30 (on the right axis), as well as age at first cohabitation, first birth, and last birth, for India as a whole and across regions. While India has experienced rapid fertility decline, there have been only slow changes in family formation and the initiation of childbearing. Across cohorts, in both the northern and southern states, the vast majority of women have married and have married young. The average age at first cohabitation was below age 20 even in the youngest cohort of women. Moreover, despite substantially lower fertility in southern states compared to northern states, women in both regions started their childbearing careers before age 22, on average. Lower fertility in India’s southern states is not substantially explained by postponement of childbearing.

Regional and cohort differences are, however, accompanied by differences in the timing of last birth. Across cohorts in India the average age at last birth has declined substantially, from 32.1 to 25.7. Although women across cohorts and regions initiated childbearing at similar ages, more recent cohorts and women in southern states ended their childbearing careers at younger ages.

How have women in India ended their childbearing careers at younger ages? The country once again stands out as a global outlier in its reliance on sterilization, a procedure obtained by 38% of married women ages 15‒49. The next most common form of contraception, condoms, is only used by 9% of married women.

**Figure 5:**
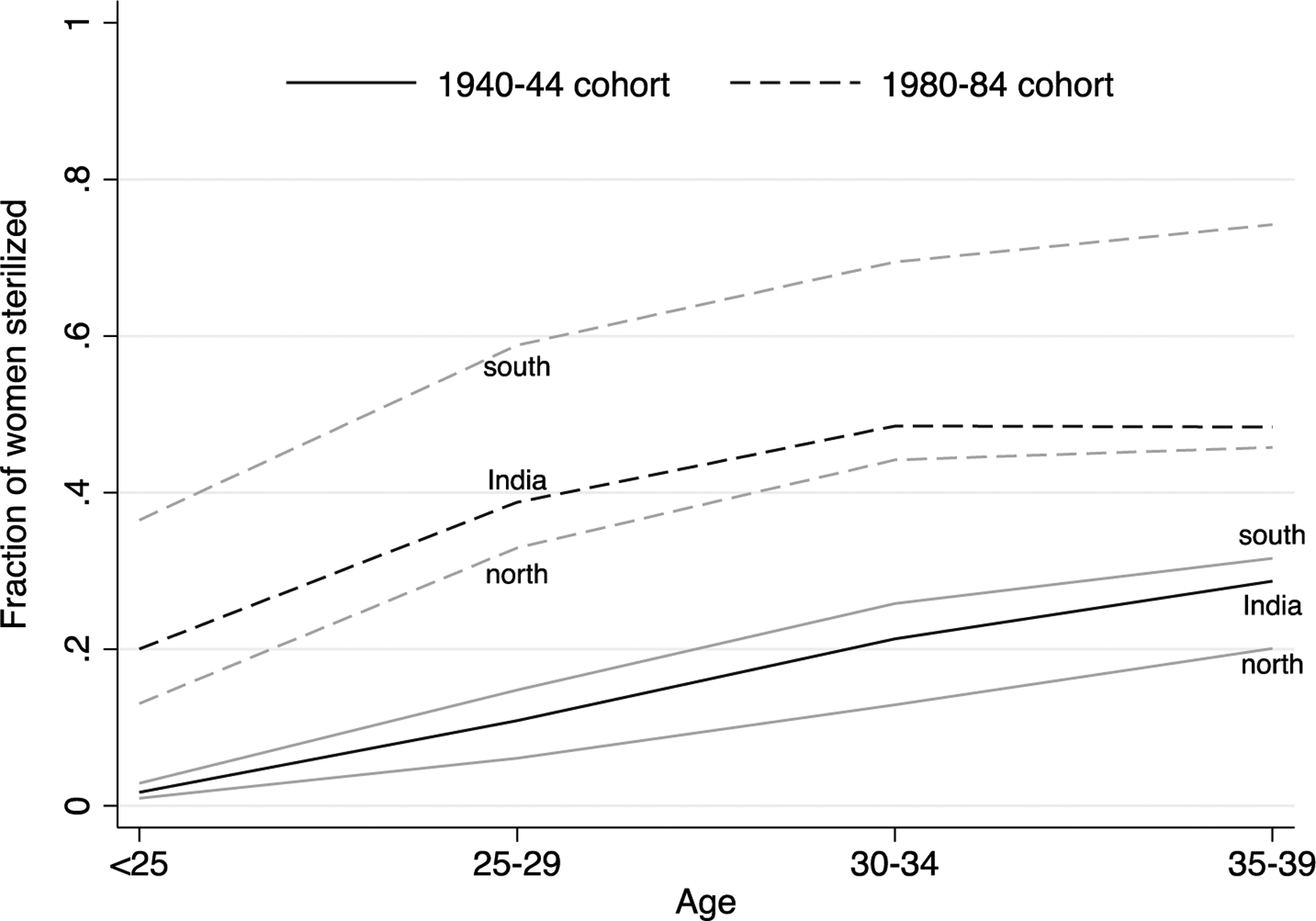
Female sterilization increasingly common among younger cohorts in India *Note*: The figure shows the fraction of women who obtained a sterilization, by age. Solid lines show estimates for the 1940‒1944 cohort. The dashed lines show estimates for the 1980–1984 cohort. For each cohort, the gray lines are estimates for the southern and northern states. *Source*: NFHS 1‒5.

Figure 5 shows that about half of Indian women in the 1980‒1984 cohort obtained a sterilization by age 35, up from about one-fifth in the 1940‒1944 cohort. In the southern states about 70% of the younger cohort ended their fertility via sterilization by this age. In results not shown, sterilization after the birth of a second or higher order child has become increasingly common.

## 4. Discussion

In this article, we join others who have examined global outliers in the fertility transition in order to illuminate alternate pathways to fertility decline (Bryant 2007; Bongaarts 2017; Bongaarts and Casterline 2013). India’s fertility transition is important because India represents a large fraction of the global population, and because of what it can teach demographers about the conditions for low fertility more generally. Although there is significant heterogeneity across Indian states, fertility is declining nationwide and has reached sub-replacement levels through mechanisms that differ from both LMICs and HICs in several key ways. Women in India continue to marry and start having children at young ages. Fertility has declined across cohorts as women end their childbearing careers at younger ages. Terminating childbearing via sterilization is a growing trend, with about half of women among younger cohorts doing so by age 35.

The distinctive pattern of fertility decline that has emerged in India, absent any dramatic changes in the timing of family formation or female labor force participation, suggests that this decline is motivated by factors that differ from those in other countries. Unfortunately, our analysis is subject to limitations that preclude us from separating the external influence of the state versus individual preferences for fewer children. In India, the dearth of opportunities for young women in higher education and the labor force means that these years are instead largely devoted to family formation and childbearing. Research on India and other countries suggests that couples may be motivated to limit their fertility in order to make greater investments in each individual child (Bryant 2007; Visaria 2022).

Alternatively, India has a documented history of coercive and forced sterilization (Gupte 2017; Nandagiri 2022), which could also be a driver of fertility decline. Unfortunately, we are unable to account for women’s motivations for and attitudes towards sterilization. Given India’s fraught history and the increasing rate of female sterilization, more research is needed on women’s desire for sterilization, as well as alternate methods of contraception.

Now the most populous country in the world, India has attained sub-replacement fertility through pathways that differ from HICs. This suggests that there is not simply one pathway to demographic transition. Although the link between women’s socioeconomic behaviors and fertility is well-documented, fertility decline may still occur in the absence of a delay in family formation, high female labor force involvement, or access to a wide range of contraceptive methods.
